# Health insurance and healthcare utilisation for Shenzhen residents: a tale of registrants and migrants?

**DOI:** 10.1186/1471-2458-12-868

**Published:** 2012-10-12

**Authors:** Kelvin KF Lam, Janice M Johnston

**Affiliations:** 1 Department of Community Medicine, School of Public Health, The University of Hong Kong, 5/F, William MW Mong Block, Faculty of Medicine Building, 21 Sassoon Road, Hong Kong, China

**Keywords:** Health insurance coverage, Healthcare utilisation and migrant

## Abstract

**Background:**

Shenzhen’s rapid growth and urbanisation has attracted a large, mobile, migrant working population. This article explores health protection through the means of social health insurance between migrants and registrants and their point of access to healthcare.

**Methods:**

A cross-sectional questionnaire survey was conducted in Shenzhen, with a random sample of 793 registered and 750 non-registered residents. Chi-square test and multivariate logistic regression were applied to analyse the association between health insurance coverage with Hukou registration status and healthcare utilisation.

**Results:**

Amongst 1543 respondents, 43.1% of non-registered residents were uninsured. Being non-registered strongly predicted for no insurance (OR = 5.00; CI 3.53,7.07) and have purchased additional/ private insurance (OR = 2.99; CI 1.66,5.37). Migrants who self-reported chronic health conditions were also more likely to utilise health services in general (OR = 2.77; CI 1.18,6.52).

**Conclusions:**

Inadequate health insurance coverage for migrants as observed in Shenzhen remains a challenge for the Chinese health reform. Our results suggest that the current insurance system must seek to include migrants in order to achieve universal coverage and improved health protection for its population.

## Background

Since the 1979 economic reform, rural workers in China have migrated to urban areas and Special Economic Zones for improved employment opportunities and living conditions. Approximately 16% of the current 1.3 billion Chinese population is recognised as the ‘floating or migrant population’ [1]. Shenzhen, the first Special Economic Zone established by the central government, currently hosts 12 million migrant workers, a staggering 86% of its total population [2]. The current ‘Hukou’ registration system in China entitles those who are registered with the local government to benefit from its social welfares, including access to labour employment and healthcare insurance schemes. Migrant workers in Shenzhen who are registered with a non-local Hukou, i.e. from their original place of residence, are not entitled to these benefits. The temporary residence card (TRC) permits migrants to receive selected welfares but many migrants still lack the TRC and are regarded as ‘unregistered’ [3].

In the past decade, several major reforms in the healthcare financing system have been implemented in an effort to overcome rising health expenditures and disparities in health status, particularly across the urban–rural divide [4-6]. This included the introduction of social medical insurance schemes such as the Urban Resident-based Basic Medical Insurance (URBMI), the Urban Employee Basic Medical Insurance (UMBMI) and the New Rural Cooperative Medical Insurance Scheme (NRCMS). Although one of the main reform objectives was to achieve over 90% health insurance coverage by 2010 [7], health insurance coverage for migrants remains a challenge for the lack of access through the Hukou restrictions. Additional/ private insurance provides an alternative to social insurance, but such schemes are often offered at a premium, subject to adverse selection and have restricted coverage for severe illnesses that require hospitalisation [8].

Population migration is associated with increased risk for communicable, non-communicable, and occupational diseases, mental health disorders, and health disparities [9-12], as well as poorer socio-economic status and worse living conditions [13]. As migrants have less access to healthcare insurance and fewer financial resources than local registrants, high costs of healthcare may have catastrophic consequences. The lack of health insurance restricts (for financial reasons) patient’s access to care thus potentially denying them treatment at an appropriate level of care. Conversely, moral hazard deriving from private insurance coverage may lead to the inefficient allocation of resources for tertiary care services [14].

A direct relationship between the range of services, price, and quality of care exists across the three tier healthcare structure in China (Table 1), with the majority of resources and manpower focused on tier 3 facilities and vice-versa [15,16]. Even though tier 1 facilities are more affordable, these community centres are not designed to cater for more advanced medical procedures that typically require hospitalisation. Migrants, particularly for those uninsured or with self-reported chronic health conditions, may not utilise beyond tier 1 facilities due to affordability [17].

**Table 1:** Source of healthcare service utilisation by the Chinese three-tier healthcare system

Tier 1-	community health centre and private clinics;
Tier 2-	district hospitals, county hospitals and private hospitals;
Tier 3-	municipal hospitals, provincial hospitals, military hospitals and University affiliated hospitals.

The objective of this study is to identify the associations between 1) health insurance coverage (both social and private insurance) by Hukou registration status (registrants with the local Shenzhen Hukou vs. migrants who lack local Hukou registration), 2) healthcare utilisation rates in general by Hukou registration and 3) point of access for healthcare services in respect to the three-tier healthcare structure and health insurance status; in Shenzhen, Guangdong, People’s Republic of China.

## Methods

### Sampling

This cross-sectional telephone survey targeted all households with a residential telephone number in Shenzhen. Based on a hypothesized difference in the proportion of residents and migrants with health insurance coverage of 0.5 (80% power and alpha of 0.05), we estimated a sample size of 1000 respondents was required. The estimatedw?> sample was increased to 1500 respondents to enable subgroup analysis (4 subgroups).

### Subject recruitment

**Figure 1:**
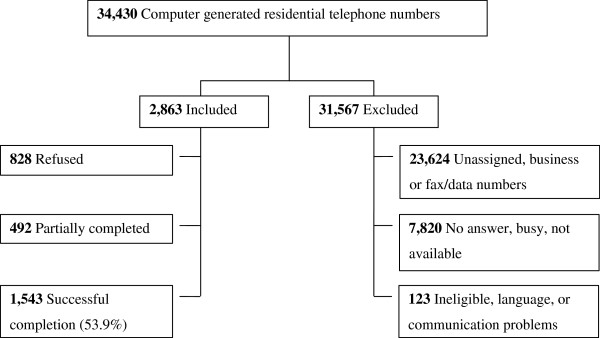
Subject recruitment flow chart.

Telephone numbers were computer generated by combining the local residential prefix (+86 755) to a complete randomly generated 8-digits sample. A total of 34,430 residential numbers were randomly generated (Figure 1). Each residential number was dialed a minimum of 3 times, at different times of the day and different days of the week. Upon successful contact, household members aged 18 years or above, who spoke Cantonese, Mandarin or English were recruited for the interview. If more than one qualified household member was available, the individual with the nearest birthday to the date of the telephone call was recruited. The survey was conducted in May 2010 by trained and experience telephone researchers from the Social Sciences Research Centre at the University of Hong Kong.

### Survey instrument

The questionnaire contained 65 questions that were divided into five sections: the first section collected basic socio-demographics, the second section assessed health condition and risky behaviour, the third section on health seeking behaviours, the fourth section on doctor consultations, and the fifth section on hospital admissions. Questions were formulated and compiled after we reviewed similar reports in the literature [18] on current policy directions, the prevailing characteristics of the Shenzhen health care system as well as drawing specific questions from the Thematic Household Survey in 2002 [19], and the 4th National Health Census [20]. All questions were piloted for comprehensibility, face and content validity on an independent sample (n = 5) of research postgraduate students with personal knowledge of the health care system in the Peoples Republic of China. Minor adjustments were made to the questionnaire after the pilot study.

Data was collected for socio-demographic characteristics (age, gender, marital status [single, married, widowed/separated or divorced], Hukou registration status [registered with a Shenzhen Hukou (temporary or permanent), not registered with a Shenzhen Hukou (migrant)], occupation [executive, professional, managerial, ordinary worker and unemployed], monthly income [sixteen categories between no income and ¥30,000 or above], self-reported chronic health conditions as diagnosed by a Western medicine practitioner [presence of chronic disease or not], age-comparative perceived health status measured on a 5-point Likert scale [1 = much better, 2 = better, 3 = about the same, 4 = worse & 5 = much worse] [21], experience of any health problems (e.g. discomfort, illness or injury) in previous 30 days [yes, no], health service utilisation [out-patient doctor consultations in the past 30 days and in-patient hospital admissions in the past 12 months], point of access for healthcare [tier 1 to tier 3 facilities] [15] and insurance status [uninsured or insured by social insurance schemes or/and additional private insurance schemes].

Verbal informed consent was obtained from all study subjects. Ethics approval was obtained from the Institutional Review Board of the University of Hong Kong/ Hospital Authority Hong Kong West Cluster (UW 10–154).

### Data analysis

The primary outcome variables were health insurance status, healthcare utilisation by Hukou registration status and source of care. For analysis, insurance status was grouped into four investigator defined categories (Table 2) and health care utilisation by either doctor consultation in the previous 30 days or hospital admission in the previous and 12 months.

**Table 2:** Defined four health insurance categories

	
Uninsured-	individuals who has neither social medical insurance* or additional/ private health insurance;
Basic MIS only-	individuals who has any social medical insurance only (regardless of which social medical insurance scheme) but no additional/ private health insurance;
Additional MIS/ private insurance only-	individuals who has any additional/ private medical insurance** only;
Both basic and additional-	individuals who has both any social medical insurance and private health insurance

*Social medical insurance delineates: Urban Resident-based Basic Medical Insurance (URBMI), Urban Employee Basic Medical Insurance (UMBMI) or the New Rural Cooperative Medical Insurance Scheme (NRCMS).

**Additional/ private insurance delineates any other insurance schemes other than the social medical insurance schemes described above.

Multiple imputation through bootstrapping from a full Bayesian predictive distribution was used to impute missing values in the demographic and socio-economic variables [22]. Predictive mean matching on binary, categorical, and continuous variables was not required and missing values in the outcome variables were not imputed [23]. Missing variables were imputed ten times and subsequent regressions in analysis were consolidated by the ‘micombine’ function [24].

Marital status was dichotomised (single/separated/widowed and married) for analysis. Income categories were constructed by quintiles, low (<20%), “middle” (20-60%) and “high” (>60%). Age-comparative perceived health status into better (much better and better) and worse (about the same, worse and much worse); smoking status into current smoker (current and past smoker) and never smoker (never smoked) and alcohol drinking patterns into regular drinker (current and past drinker) and never drinker (never drinks) were dichotomised for analysis. Source of healthcare was grouped into three tiers according to the Chinese healthcare system (Table 1).

All sample data were weighted to achieve population representation using standard methodologies against the 4^th^ National Health Census data for Shenzhen.

Logistic regression models were used to estimate the crude and adjusted odds ratio and the 95% confidence intervals controlling for covariates including age, gender, marital status, local registration, occupation, education, monthly income, age-comparative perceived health status, self-reported chronic health condition, smoking and drinking behaviour, self-reported sickness in the past 30 days and point of healthcare access.

Multiple imputations were performed in R (R Foundation) Version 2.13 and regression analysis was conducted in Stata (StataCorp) Version 10.

## Results

Among 2863 valid residential telephone numbers, 1543 (response rate 53.9%) subjects completed the survey (Figure 1). The overall weighted sample of respondents had similar socio-demographic characteristics to the Shenzhen census except for educational attainment. Migrants, in contrast to registrants, were younger, single, with lower education and income, and fewer had health insurance coverage (Table 3).

**Table 3:** **Subject characteristics and crude odds ratio for being a migrant (n**_**raw**_ **= 1543; n**_**weighted**_ **= 3681)**

	**Shenzhen Study Population**	**4th National Census -Shenzhen city**	**Effect size**^**4**^	**Registered**	**Migrants**	
	**(weighted)**	**(N = 3,681)**		**(n = 793)**	**(n = 750)**	**Migrant Status**
	**%**^**3**^	**%**^**3**^		**n (%)**	**n (%)**	**Crude OR (95% C.I.)**
Female ^1^	50.4	50.7	**0.01**	422 (53.2)	356 (47.5)	0.80* (0.65, 0.99)
Age group (years) ^1^						
18-24	13.9	13.9	**0**	120 (15.1)	166 (22.1)	1
25-34	32.3	32.3		263 (33.2)	319 (42.5)	0.88 (0.66, 1.17)
35-44	24.5	24.5		224 (28.2)	156 (20.8)	0.51*** (0.37, 0.69)
45-54	14	14		99 (12.5)	46 (6.1)	0.34*** (0.23, 0.52)
55-64	8.3	8.3		44 (5.5)	31 (4.1)	0.53* (0.32, 0.89)
65+	7.1	7		30 (3.8)	22 (2.9)	0.55* (0.30, 0.99)
Marital Status ^1^						
Single/separated/widowed	25.6	19	**0.17**	198 (25.0)	278 (37.1)	
Married	74.4	81		595 (75.0)	472 (62.9)	0.54*** (0.43, 0.67)
Education Attainment ^1^						
Primary or below	10.3	17.6	**0.43**	44 (5.5)	72 (9.6)	1.22 (0.80, 1.87)
Middle/high school	46.5	57.4		319 (40.2)	412 (54.9)	1
College or above	43.2	25		430 (54.2)	266 (35.5)	0.47*** (0.37, 0.58)
Occupation ^1^						
Executive/professional/managerial	-	-		148 (18.7)	76 (10.1)	0.40*** (0.29, 0.54)
Ordinary worker	-	-		378 (47.7)	468 (62.4)	1
Unemployed	35.3	39.7	**0.07**	267 (33.7)	206 (27.5)	0.64*** (0.50, 0.82)
Monthly income ^2^						
No income	27.2	-		201 (25.3)	189 (25.2)	1.08 (0.83, 1.42)
Low income	14.8	-		72 (9.1)	130 (17.3)	1.83*** (1.29, 2.59)
Middle income	43.3	-		341 (43.0)	343 (45.7)	1
High income	14.7	-		179 (22.6)	88 (11.7)	0.42*** (0.29, 0.59)
Insurance status						
Uninsured				97 (12.2)	304 (40.5)	1
Basic MIS only				422 (53.2)	275 (36.7)	0.17*** (0.13, 0.23)
Additional MIS only				25 (3.2)	66 (8.8)	0.82 (0.48, 1.41)
Basic + Additional MIS				212 (26.7)	98 (13.1)	0.13*** (0.09, 0.18)
Registered	53.3	-		-	-	

Note: C.I. denotes lower and upper limit of 95% confidence interval.

^1^ Categories were constructed to be compatible with published census figures.

^2^ Income categories are constructed by quintiles, where “low” income denotes the first quintile (<20%), “middle” income denotes between second and fourth quintile (20-60%) and “high” income denotes fifth quintile or above (>60%).

^3^ The proportions may not add up to 100% due to rounding.

^4^ Effect size is a statistical measure that indicates the similarity of the sample compared to the underlying population, where an effect size of 0.1 is considered “small”, 0.3 “medium” and 0.5 “large”.

Statistical significance of p-values are denoted by * (p < 0.05), ** (p <0.01) & *** (p < 0.001).

Migrants were more likely to be current smokers (OR = 1.32; CI 1.04,1.67) and less likely to self-report a chronic health condition (OR = 0.48; CI 0.34,0.67) or a hospital admission in the previous 30 days (OR = 0.51; CI 0.29,0.89). Being a migrant is associated with less likelihood to attend municipal or provincial hospitals (OR = 0.45; CI 0.32,0.61) compared with health utilization at community level (Table 4).

**Table 4:** **Crude odds ratios for self-reported health status, lifestyle behaviours and health service utilisation for being a migrant (n**_**raw**_ **= 1543)**

	**Registered (n = 793)**	**Migrants (n = 750)**	**Migrant Status**
	**n (%)**	**n (%)**	**Crude OR (95% C.I.)**
Age-comparative perceived health status (better)^1^	311 (39.5)	329 (44.9)	1.00 (0.66, 1.52)
Smoking status - current smoker^2^	202 (25.5)	239 (31.9)	1.32** (1.04, 1.67)
Alcohol drinker pattern - regular drinker^2^	564 (71.1)	547 (72.9)	1.01 (0.80, 1.28)
Self-reported chronic health conditions ^3^	119 (15.0)	63 (8.4)	0.48*** (0.34, 0.67)
Health problem in previous 30 days^3^	155 (19.5)	125 (16.7)	0.83 (0.63, 1.10)
Doctor consultation in previous 30 days^3^	53 (6.7)	37 (4.9)	0.67 (0.43, 1.06)
Hospital admission in previous 12 months^3^	38 (4.8)	22 (2.9)	0.51* (0.29, 0.89)
Source of healthcare service utilitsation^4^			
Tier 1 (Community health centre and private clinics)	128 (16.1)	159 (21.2)	1
Tier 2 (Regional hospital)	134 (16.9)	159 (21.2)	0.98 (0.69, 1.38)
Tier 3 (Municipal/ Provincial hospital)	291 (36.7)	168 (22.4)	0.45*** (0.32, 0.61)

Note: C.I. denotes lower and upper limit of 95% confidence interval.

^1^Age-comparative perceived health status was dichotomised (reference = worse).

^2^Smoking status and alcohol drinking pattern were dichotomised (reference = never smoker/drinker).

^3^Variables were dichotomised chronic health conditions (reference = absence of disease), health problems in previous 30 days (reference = none), doctor consultation in previous 30 days (reference = none), hospitalisation in previous 12 months (reference = none).

^4^Source of healthcare service utilisation was segregated into three tiers (Table 1) (reference = tier 1).

Statistical significance of p-values are denoted by * (p < 0.05), ** (p <0.01) & *** (p < 0.001).

Table 5 shows strong associations between insurance coverage and Hukou registration status. After adjustment, migrants were more likely to be uninsured (OR = 5.00; CI 3.53,7.07) or have Additional MIS only (OR = 2.99 ,CI 1.66,5.37). Migrants and those unemployed were associated with lack of Basic MIS only coverage (OR_migrant_ = 0.45; CI 0.35,0.58 vs. OR_unemployed_ = 0.67; CI 0.50,0.90). Migrants who lack Basic MIS coverage may have contributed to the strong association of purchasing Additional MIS, but the same effect was not observed for the unemployed (OR_migrant_ = 2.99; CI 1.66, 5.37 vs. OR_unemployed_ = 1.28; CI 0.72, 2.28). For those who purchased Additional MIS only, in contrast to those uninsured, were far more likely to have a hospital admission in the previous 12 months (OR_Additional MIS only_ = 2.75; 1.07-7.09 vs. OR_uninsured_ = 0.44; CI 0.21,0.93). The source of healthcare service utilisation is not associated with insurance status except for registrants with Basic + Additional MIS coverage who are more likely to use Municipal/ Provincial hospitals (tier 3) (OR = 1.61; CI 1.04,2.49). Self-reported chronic health conditions did not predict insurance status.

**Table 5:** Factors predicting health insurance uptake adjusted for Hukou registration status

	**Uninsured (n = 401)**		**Basic MIS only (n** **= 697)**		**Additional MIS only (n** **= 91)**		**Basic + Additional MIS(n = 310)**	
	**Adjusted OR**		**Adjusted OR**		**Adjusted**		**Adjusted**	
	**(95% C.I.)**		**(95% C.I.)**		**OR (95% C.I.)**		**OR (95% C.I.)**	
Hukou registration status (migrant)^1^	5.00***	(3.53,7.07)	0.45***	(0.35,0.58)	2.99***	(1.66,5.37)	0.50***	(0.36,0.68)
Occupation								
Executive/ professional/ managerial	0.52*	(0.29,0.94)	0.99	(0.69,1.43)	0.46	(0.17,1.22)	1.29	(0.88,1.91)
Ordinary worker	1		1		1		1	
Unemployed	2.58***	(1.76,3.79)	0.67***	(0.50,0.90)	1.28	(0.72,2.28)	0.68*	(0.47,0.99)
Self-reported chronic health conditions ^2^	1.07	(0.65,1.77)	1.21	(0.83,1.77)	0.60	(0.24,1.48)	0.79	(0.49,1.26)
Doctor consultation in previous 30 days^2^	0.94	(0.52,1.70)	0.99	(0.61,1.60)	0.52	(0.16,1.70)	1.13	(0.60,2.12)
Hospital admission in previous 12 months^2^	0.44*	(0.21,0.93)	1.19	(0.66,2.17)	2.75*	(1.07,7.09)	1.04	(0.48,2.26)
Source of healthcare service utilitsation^3^								
Tier 1 (Community health centre a nd private clinics)	1		1		1		1	
Tier 2 (Regional hospital)	0.74	(0.48,1.14)	1.09	(0.76,1.56)	0.79	(0.39,1.58)	1.38	(0.87,2.19)
Tier 3 (Municipal/ Provincial hospital)	0.73	(0.48,1.12)	0.98	(0.70,1.37)	0.74	(0.36,1.53)	1.61*	(1.04,2.49)

Note: Regression also adjusted for gender, age, marital status and education.

C.I. denotes lower and upper limit of 95% confidence interval.

^1^ Registration status was dichotomised (reference = registered).

^2^Variables were dichotomised chronic health conditions (reference = absence of disease), health problems in previous 30 days (reference = none), doctor consultation in previous 30 days (reference = none), hospitalisation in previous 12 months (reference = none).

^3^Source of healthcare service utilisation was segregated into three tiers (Table 1) (reference = tier1).

Statistical significance of p-values are denoted by * (p < 0.05), ** (p <0.01) & *** (p < 0.001).

Table 6 shows factors predicting healthcare utilisation stratified by Hukou registration status. After adjustment, self-reported chronic health conditions was a strong predictor for utilisation in both migrants and registrants, (OR_migrants_ = 2.77; 1.18,6.52 vs. OR_registrants_ = 3.29; 1.84,5.89). Insurance status did not predict healthcare utilisation by registration status.

**Table 6:** Factors predicting healthcare utilisation stratified by Hukou registration status

	**Migrants**		**Registrants**	
	**Adjusted OR**		**Adjusted OR**	
	**(95% C.I.)**		**(95% C.I.)**	
Female	1.80	(1.00, 3.26)	1.24	(0.74, 2.07)
Age	0.98	(0.95, 1.01)	1.00	(0.97, 1.02)
Marital status				
Single/separated/widowed	1		1	
Married	0.90	(0.44, 1.86)	0.72	(0.38, 1.36)
Occupation				
Executive/ professional/ managerial	0.69	(0.23, 2.04)	1.82	(0.93, 3.55)
Ordinary worker	1		1	
Unemployed	0.94	(0.47, 1.87)	1.49	(0.83, 2.67)
Education				
Primary or below	0.74	(0.22, 2.41)	2.85**	(1.11, 7.31)
Middle/high school	1		1	
College or above	0.63	(0.33, 1.20)	1.34	(0.77, 2.34)
Self-reported chronic health conditions ^3^	2.77*	(1.18, 6.52)	3.29**	(1.84, 5.89)
Insurance status				
Uninsured	1		1	
Basic MIS only	1.40	(0.70, 2.80)	1.14	(0.48, 2.74)
Additional MIS only	1.00	(0.32, 3.11)	2.64	(0.76, 9.16)
Basic + Additional MIS	1.55	(0.65, 3.72)	1.35	(0.50, 3.62)

Note: C.I. denotes lower and upper limit of 95% confidence interval.

Statistical significance of p-values are denoted by * (p < 0.05), ** (p <0.01) & *** (p < 0.001).

## Discussion

This study has shown that in a city with a predominantly migrant population, inequality in the distribution of social health insurance remains a challenge. Health insurance coverage in a quasi-Bismarck insurance model like China can be used as a proxy of health protection for its population. Achieving universal health coverage for the world’s largest population is no doubt unparalleled in scale and unprecedented; some Chinese cities such as Shanghai [25], Shantou [26] and Xiamen [27] have already reported near 100% coverage, but others such as Beijing [28] and Zhuhai [26] report only 6% and 2% rural participation respectively. Some local governments have introduced pilot medical insurance schemes- e.g. Medical Insurance System for Migrant Employees (MISM) to redress this inequity, however compliance has been poor with few migrants enrolled [10]. Under the current Hukou system, migrant workers will inevitably find it difficult to access government benefits such as healthcare. This evidence supports policy interventions or indeed the Hukou reform to improve on the transferability and portability of insurance schemes across cities in China, as found in other countries such as Canada [29] with large mobile populations. However, policymakers must also consider the larger social issues from rapid urbanization such as economic gains, societal harmony, the role of both local and central governments in providing housing, education, pension or employment and the financial impact on fiscal reserves from welfare expenses.

The outcomes of this study, lower socioeconomic status, higher rates of self-reported chronic health conditions, increased uptake of Additional MIS insurance and a possible preference for community centres or private clinics (tier 1 facilities) among migrant workers are similar to that found in the literature [30-32]. However the choice for community centres or private clinics may be mitigated by affordability, health care policy-guided behaviors or perhaps reflects of the effectiveness of these insurance policies themselves. While self-reported chronic health conditions were not associated with health insurance uptake, it was strongly associated with healthcare utilisation in both registrants and migrants. The Hukou system, for similar reasons explained above, is currently preventing migrant patients acquiring free services from non-registered cities, curbing potential exploits and abuse; but resulting in an unethical consequence of restricting patients to their hometowns through denying them medication elsewhere.

In our study, hospital admissions were associated with migrants who had additional MIS coverage only. Although not significant, these patients appear to be less likely to utilize tier 2 and tier 3 hospitals. This was unexpected as health service utilisation among the whole sample was driven by chronic health conditions. Still, it has been reported elsewhere that illegal private clinics may offer more affordable health care service for migrants [33], a consequence of which may be poorer health outcomes and supply side moral hazard. From the unpublished data in the same study, more migrants relied on self-medication when reported a problem compared with registrants, and this suggests migrants may increasingly resort to self-medication, perhaps in the form of Traditional Chinese Medicine, instead of seeking health professionals for care.

### Limitations of this study

Although information on the exact type of social health insurance scheme was obtained from every respondent, we did not further stratify the various social health insurance schemes for analysis. The compression of social insurance schemes for analysis may obscure some of the marginal differences in outpatient and inpatient visits due to different reimbursement compositions. Nevertheless, our aim of the study was to investigate the availability of health protection through social health insurance and additional/ private insurance, but not the impact of different health insurance schemes on health protection, however other studies have already began to inquest such differences [34]. The migrants in our study were relatively ‘healthy’ with fewer self-reported chronic health conditions and better comparative self-rated health status, which is not consistent with health problems amongst migrants as reported in wider literature [30]. We suggest the ‘healthy migrant’ effect may exist in Shenzhen, where the generally younger population are subject to later onset of chronic symptoms, hence delayed diagnosis of chronic illness. The inherent limitation in a cross-sectional study means we can only infer associations rather than causality; and the ‘cold-calling’ nature in telephone surveys has resulted in a less than desirable response rate which may contribute to selection bias. However, the response rate is comparable with other published studies using questionnaire designs. Although our sample was randomised by telephone numbers, migrants who are clustered in factory dormitories or in rental places may lack permanent landline numbers. These unrepresented individuals may differ systematically and may contribute to selection bias.

## Conclusions

Despite visions by the Chinese government to achieve universal health insurance coverage by 2012 as part of its health reform target, migrant workers appear to be neglected from its social health insurance net.

Special Economic Zones like Shenzhen, which is constituted almost entirely by migrant workers, should seek to pioneer insurance reforms from the local government level to extend health protection for its migrant population. Efforts should be focused on evaluating the eligibility of insurance schemes for both its registered and non-registered residents, improve the portability and transferability of schemes through collaboration within the Guangdong province or other major origins of migrant workers and broaden insurance reimbursements to extend access to healthcare beyond tier 1 facilities.

Providing health insurance to migrant workers is of paramount importance in maintaining a balanced and healthy workforce for the continual economical development in China.

## Abbreviations

URBMI: Urban Resident-based Basic Medical Insurance; UMBMI: Urban Employee Basic Medical Insurance; NRCMS: New Rural Cooperative Medical Insurance Scheme; MISM: Medical Insurance System for Migrant Employees.

## Competing interests

The authors declare that they have no competing interests.

## Authors’ contributions

KL conducted the literature review, performed the statistical analysis and prepared the manuscript. JJ conceived the study and gave careful advice and revised the manuscript. All authors read and approved the final manuscript.

## Pre-publication history

The pre-publication history for this paper can be accessed here:

http://www.biomedcentral.com/1471-2458/12/868/prepub
